
JOURNAL INFORMATION
==============================
NLM Title Abbreviation: Wirtschaftsdienst
Journal ID: Wirtschaftsdienst
NLM Journal ID: 101086612

Wirtschaftsdienst (Hamburg, Germany : 1949)

ISSN: 0043-6275

ARTICLE INFORMATION
==============================
PMCID: PMC8287293
PMID: 34305187
DOI: 10.1007/s10273-021-2955-9
Article ID: 2955
Article version: 1
Subjects: Zeitgespräch

Wie kann Wirtschaftspolitik zur Eindämmung des Populismus beitragen?

Gold Robert Robert.Gold@ifw-kiel.de

grid.462465.7 0000 0004 0493 2817 Innovation und internationaler Wettbewerb, Institut für Weltwirtschaft (IfW), Kiellinie 66, 24105 Kiel, Deutschland
Electronic publication date: 2021 Jul 19
Print publication date: 2021
Volume: 101
Issue: 7
First page: 500
Last page: 504
Copyright: © Der/die Autor:in(nen) 2021
License: Open Access: Dieser Artikel wird unter der Creative Commons Namensnennung 4.0 International Lizenz veröffentlicht (https://creativecommons.org/licenses/by/4.0/deed.de).
Open Access wird durch die ZBW — Leibniz-Informationszentrum Wirtschaft gefördert.
License URL: https://creativecommons.org/licenses/by/4.0/

issue-copyright-statement: © The Author(s) 2021

==============================

Die Pandemie hat die Schwächen populistischer Politik schonungslos offenbart. Statt sich am Stande der Wissenschaft zu orientieren und die Bevölkerung über geeignete Maßnahmen zum Schutz gegen das Coronavirus zu informieren, haben sich populistische Politiker:innen durch ihre Ignoranz wissenschaftlicher Fakten und ihre Orientierung an Befindlichkeiten statt an Notwendigkeiten hervorgetan. Die Folge war eine geringere Akzeptanz von Infektionsschutzmaßnahmen und ein Anstieg der Infektionszahlen. Das teilweise desaströse Pandemiemanagement hat der Popularität von Populisten jedoch erstaunlich wenig Abbruch getan. Mehr als 74 Mio. Amerikaner:innen stimmten für eine zweite Amtszeit von Donald Trump. Die Zustimmung zu den Tories unter Boris Johnson scheint ungebrochen. In allen europäischen Ländern haben populistische Parteien mittlerweile Fuß gefasst (vgl. Abbildung 1) und ihr elitenfeindlicher, faktenverdrehender und an „Volkes Wille“ orientierter Politikstil hält Einzug in den politischen Mainstream.

Natürlich gehört es zum demokratischen Wettbewerb, dass neue Parteien auftreten, wenn hinreichend viele Wähler:innen ihre Interessen durch die etablierten Parteien nicht vertreten sehen. Tatsächlich geht der Aufstieg

* Dieser Aufsatz basiert auf einer am 25. Mai 2021 auf der Tagung des Forums for a New Economy vorgestellten Studie „From a better understanding of the drivers of populism to a new political agenda” im Auftrag des Forums for a New Economy.

Abbildung 1

**Stimmanteile nationalistischer Parteien bei Europawahlen**

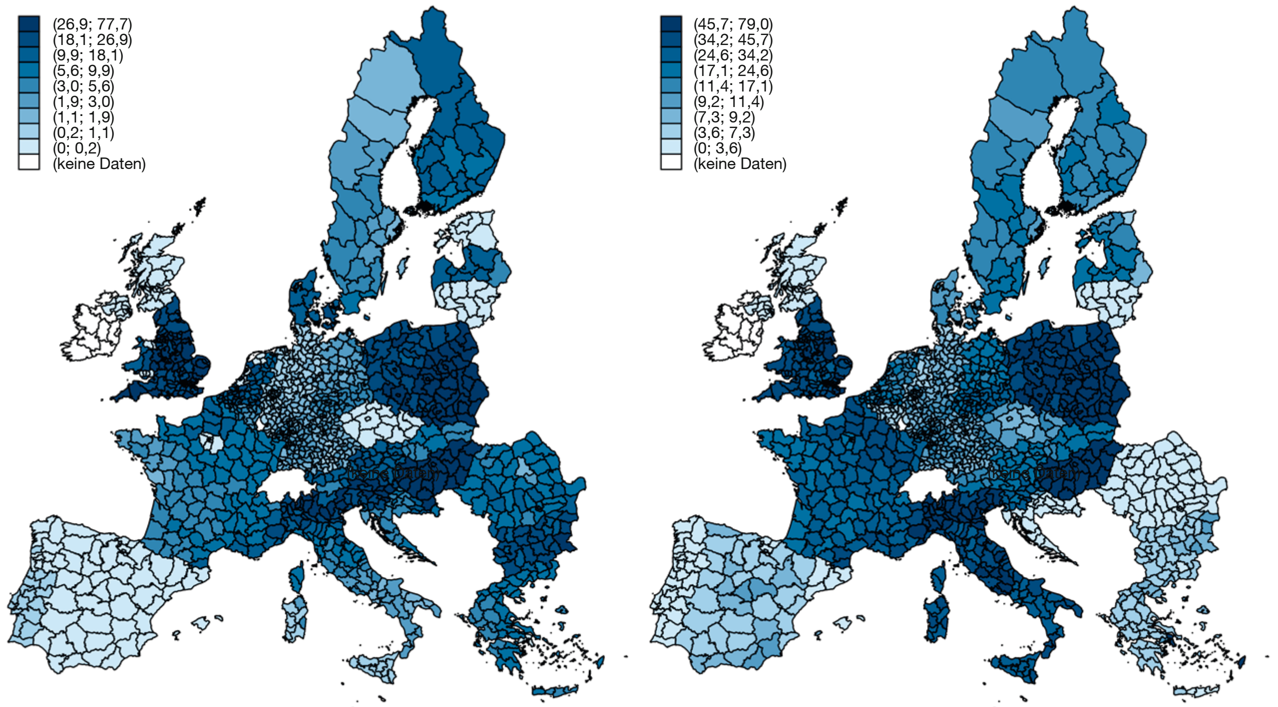

2009 – Ø 10,18 %

2019 – Ø 20,69 %

Anmerkungen: Die Grafik zeigt Stimmanteile nationalistischer Parteien bei den Wahlen zum europäischen Parlament 2009 (links) und 2019 (rechts) auf NUTS3-Ebene. In Kroatien wurde die Wahl 2009 im Jahr 2013 nach dem Beitritt zur EU nachgeholt.

Quelle: Daten aus Gold und Lehr (2021).

des Populismus einher mit einer Zunahme der Wahlbeteiligung. Offenbar gelingt es populistischen Kandidat:innen, enttäuschte Wähler:innen wieder für die politische Teilhabe zu mobilisieren. Problematisch scheint aber die einseitige Ausrichtung auf den unterstellten „Volkswillen“ und das Versprechen, diesen ohne Rücksicht auf Partikularinteressen zu exekutieren – schließlich ist die aktuell populärste Politik nicht notwendigerweise die gesellschaftlich optimale. Vielmehr erfordert eine gemeinwohlorientierte Politik, auch unpopuläre Reformen durchzusetzen – und die Wähler:innen von deren langfristigem Nutzen zu überzeugen. Populismus steht dem in zweierlei Hinsicht entgegen. Einerseits orientiert sich populistische Politik statt an Wohlfahrtssteigerungen an Meinungen und Befindlichkeiten im Status quo. Andererseits untergräbt die polarisierende Art populistischer Kampagnen die Basis für lösungsorientierte politische Debatten, die Voraussetzung dafür sind, mehrheitliche Akzeptanz für notwendige Reformen zu erzielen.

Entsprechend groß ist das Interesse der sozialwissenschaftlichen Forschung am Populismus. Seit dem Brexit-Referendum und der Wahl Donald Trumps 2016 hat sich eine Reihe von Studien mit den Ursachen von Populismus beschäftigt, wobei der Fokus auf dem Erstarken des Populismus in den westlichen Industrienationen liegt (siehe Guriev und Papaioannou, 2021; Rodrik, 2020; Fetzer und Gold, 2019). Folglich beschäftigt sich die Mehrzahl der Arbeiten mit Populismus vom rechten Rand des politischen Spektrums. Aus der Gesamtschau lassen sich bereits einige Politikimplikationen ableiten.

## Ursachen des Populismus

Die Ursachen des Populismus lassen sich grob in zwei Kategorien zusammenfassen:

- *Kulturelle Ursachen*: Die Unterstützer populistischer Parteien sehen gemeinsame Werte, Normen und Überzeugungen in Gefahr durch moderne gesellschaftliche Entwicklungen. Eng damit verbunden sind psychosoziale Faktoren wie Zukunftsängste oder Xenophobie.
- *Ökonomische Ursachen*: Die Unterstützung populistischer Parteien gilt als Indikator für eine Nachfrage nach protektionistischer, an nationalen Interessen orientierter Politik, die die Bürger:innen vor den negativen Auswirkungen globaler wirtschaftlicher Entwicklungen schützen soll.

Leider sind die Interaktionen der kulturellen und ökonomischen Bestimmungsfaktoren noch wenig erforscht. Kulturelle Ursachen gelten als Determinante der beachtlichen Unterschiede im Umfang der Unterstützung populistischer Parteien zwischen Ländern, Regionen und gesellschaftlichen Gruppen. Für den allgemeinen Trend zum Erstarken populistischer Parteien scheinen ökonomische Gründe bedeutsamer. Angebotsseitig lässt sich eine strikte Unterscheidung ohnehin kaum aufrechterhalten. Der Erfolg des Populismus beruht nicht zuletzt auf einer sehr erfolgreichen Kommunikationsstrategie, die Zusammenhänge zwischen ökonomischen Härten, Internationalisierung, Liberalisierung und Migration konstruiert. Ängste vor dem Verlust einer nationalen Identität werden so mit ökonomisch bedingten Zukunftsängsten verbunden und einer gesellschaftlichen „Elite“ angelastet, die dem reinen „Volkswillen“ angeblich zuwiderhandelt.

Jedenfalls zeigen sich Wähler:innen anfälliger für populistische Rhetorik, wenn sie den negativen Wirkungen ökonomischer Schocks ausgesetzt sind. Den Auswirkungen wirtschaftlichen Wandels lässt sich politisch deutlich einfacher begegnen als den kulturellen Ursachen des Populismus. Daher stehen die ökonomischen Ursachen des Populismus im Zentrum dieser Betrachtung. Genauer werden wirtschaftspolitische Maßnahmen erörtert, die diesen Ursachen entgegenwirken können.

## Wirtschaftspolitische Anknüpfungspunkte

Die Zunahme des populistischen Wählerpotenzials lässt sich auf makroökonomische Entwicklungen zurückführen (vgl. Abbildung 2). Die neuere Forschung hat sich besonders auf Effekte der Globalisierung der Finanzmärkte (vor allem Finanzkrisen), der internationalen Handelsintegration (vor allem Importwettbewerb aus Niedriglohnländern), des technologischen Wandels (vor allem Automatisierung und Digitalisierung) und der internationalen Migration (vor allem Immigration von Flüchtlingen) konzentriert. Während die akademische Forschung meist bestrebt ist, den Einfluss der einzelnen Effekte unabhängig voneinander zu identifizieren, wirken sie hinsichtlich ihrer politischen Konsequenzen doch zusammen.

Ökonomische Entwicklung bedingt und befeuert strukturellen Wandel. In Industriestaaten verlagert sich die Wertschöpfung in den Dienstleistungsbereich. Auch innerhalb des Verarbeitenden Gewerbes nimmt der Anteil von Jobs

Abbildung 2
**Die ökonomischen Ursachen des Populismus**

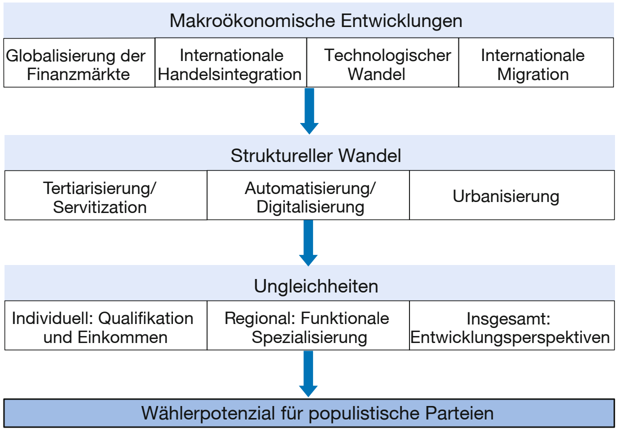

Anmerkung: Die Grafik fasst die Ergebnisse der jüngeren Forschung zu den ökonomischen Ursachen des Populismus zusammen.

Quelle: eigene Darstellung.

mit Dienstleistungscharakter zu. Gleichzeitig schreiten Automatisierung und Digitalisierung voran. Diese Entwicklungen verstärken auch den Trend zur Urbanisierung, da neue Jobs mit Servicecharakter eher in den städtischen Zentren entstehen. Nun geht wirtschaftlicher Fortschritt von jeher einher mit dem Aussterben alter Industrien und dem Aufstieg neuer, produktiverer Branchen. Wenn Arbeitnehmer:innen sektoral – und folglich regional – mobil sind, können sie von dieser Entwicklung profitieren. Auch wenn der strukturelle Wandel zu Turbulenzen führt, sorgt er doch dafür, dass Arbeitnehmer:innen anspruchsvollere, sicherere und besser bezahlte Jobs finden können. Die Wandlungsdynamik hat sich durch Globalisierung und technologischen Wandel allerdings verstärkt – und damit auch die Unsicherheit über die zukünftige wirtschaftliche Entwicklung sowie die Ungleichheiten in der Wirkung strukturellen Wandels.

Von besonderer Bedeutung sind Qualifikationsunterschiede. Während hochqualifizierte Arbeitnehmer:innen sektoral mobil sind und Lohnzuwächse verzeichnen können, nimmt die sektorale Mobilität geringqualifizierter Arbeitnehmer:innen ab. Ihre im jeweiligen Beruf erlernten Fähigkeiten werden von anderen Branchen nur bedingt nachgefragt. Folglich stagnieren die Lohneinkommen Geringqualifizierter und es fällt ihnen schwer, zusätzlich Kapitaleinkommen zu erzielen. Analog haben sich die regionalen Unterschiede in der Betroffenheit von strukturellem Wandel erhöht. Vor allem urbane Zentren profitieren von Hochlohnjobs in zukunftsorientierten Branchen. Periphere Regionen haben nicht nur mit dem Verlust an Arbeitsplätzen zu kämpfen, sondern auch mit dem Wegzug junger, hochqualifizierter und konsumfreudiger Arbeitnehmer:innen. Neben den direkten Effekten auf die regionale Wirtschaft hat dies Auswirkungen auf das soziale Leben vor Ort und verringert die Lebensqualität für die Zurückgebliebenen.

Struktureller Wandel bringt damit nicht nur relative Einkommensverluste für negativ betroffene Individuen und Regionen. Vielmehr verlieren sie Entwicklungsperspektiven. Hier liegt wohl die eigentliche politische Sprengkraft, dass die „Verlierer" des strukturellen Wandels kaum noch Chancen sehen, zu den „Gewinnern" aufzuschließen. Das erzeugt Frust und erhöht das Wählerpotenzial für populistische Parteien. Der Erfolg populistischer Parteien ist es, sich dieses Potenzial zu erschließen. Vor allem rechtspopulistische Parteien propagieren eine auf nationale Interessen konzentrierte Politik, die Schutz vor den Unbillen der Globalisierung und des strukturellen Wandels verspricht.

## Wirtschaftspolitische Maßnahmen

Die Reduktion von Ungleichheit trägt zur Stabilisierung des marktwirtschaftlichen Systems bei, indem sie protektionistischen und nationalistischen Politiken die Unterstützung entzieht. Die Forschung zu den ökonomischen Ursachen des Populismus liefert hierzu einige wirtschaftspolitische Ansatzpunkte:

### Wohlfahrtspolitik

Wohlfahrtsstaatliche Maßnahmen reduzieren Ungleichheit durch Umverteilung und federn negative Auswirkungen ökonomischer Schocks ab. Entsprechend zeigt sich im Ländervergleich, dass der Einfluss von Globalisierung und technologischem Wandel auf die Unterstützung populistischer Parteien in starken Wohlfahrtsstaaten deutlich geringer ausfällt als in schwachen (Rodrik, 2018; Glitsch, 2021). Werden wohlfahrtsstaatliche Transfers beschnitten, erhöht dies die Unterstützung populistischer Politik (Fetzer, 2019). Neben dem Einkommenseffekt dürfte hier gerade auch der Versicherungseffekt bedeutsam sein, da Populisten sehr bewusst mit Zukunftsängsten spielen.

Wohlfahrtspolitik kann also zur Eindämmung des Populismus beitragen. Sie alleine reicht aber nicht aus, die „Verlierer" des strukturellen Wandels zu kompensieren. Diese sind nicht allein von relativen Einkommensverlusten betroffen, die durch Transferzahlungen ausgeglichen werden könnten. Der Verlust an Entwicklungsperspektiven erfordert weitergehende Politikansätze, die betroffene Individuen und Regionen dabei unterstützen, sich neue Entwicklungspfade zu erschließen.

### Arbeitsmarktpolitik

Globalisierung und technologischer Wandel wirken über ihre Arbeitsmarkteffekte auf die Unterstützung populistischer Parteien (Dippel et al., 2021). Entsprechend groß ist das Potenzial, durch Arbeitsmarktpolitik den ökonomischen Ursachen des Populismus zu begegnen. Natürlich ist die Vermeidung von Arbeitslosigkeit bedeutsam, wie auch Wiedereingliederungshilfen. Der Erfolg populistischer Parteien hängt aber nicht allein von der Arbeitslosigkeit ab. Vor allem geringqualifizierte Beschäftigte in vom strukturellen Wandel betroffenen Branchen reagieren auf ökonomische Schocks, indem sie populistische Kandidat:innen unterstützen – sei es aus Sorge um die Sicherheit des Arbeitsplatzes, sei es aufgrund der abnehmenden Aufstiegschancen. Zur Eindämmung des Populismus muss Arbeitsmarktpolitik daher auch die Fort- und Weiterbildung von Menschen im Beruf fördern. Zielsetzung ist es, auch Menschen mit geringer Qualifikation in die Lage zu versetzen, von den Aufstiegschancen, die der strukturelle Wandel generiert, zu profitieren (Bode und Gold, 2018).

Es bedarf einer Fort- und Weiterbildungsoffensive, die das Schlagwort vom „Lebenslangen Lernen" mit Inhalt füllt. Die Kooperation der Arbeitgeber:innen ist hierzu unerlässlich. Sie kann durch finanzielle Anreize, regulativ, aber auch durch öffentliche Auftragsvergabe nur an Anbieter mit Qualifizierungskonzept stimuliert werden. Erhebliches Potenzial liegt außerdem in der konkreten Anwendung neuer Technologien. Künstliche Intelligenz muss menschliche Arbeit nicht ersetzen, sie kann sie auch komplementieren. Gerade Geringqualifizierte könnten ihre Produktivität steigern, wenn sie in der Anwendung intelligenter Technologien geschult werden und die Entwicklung entsprechender Anwendungen vorangetrieben wird.

### Regionalpolitik

Auch Menschen, die nicht direkt von Arbeitsmarkteffekten betroffen sind, unterstützen populistische Kandidat:innen, wenn ihre Heimatregion mit strukturellem Wandel zu kämpfen hat (Dippel et al., 2015). Die Unterschiede zwischen peripheren Regionen und Agglomerationen haben dabei nicht nur zugenommen, sie haben sich auch ausdifferenziert. Periphere Regionen hinken nicht nur in der wirtschaftlichen Entwicklung zurück, sie sind zunehmend abgekoppelt von den sozio-ökonomischen Entwicklungen, die den Erfolg urbaner Zentren verstärkt. Ein Grund dafür ist die selektive Abwanderung, die die Entwicklungspotenziale peripherer Regionen nachhaltig einschränkt.

Tradiertes Ziel der Regionalpolitik ist es, die „Konvergenz" peripherer Regionen zu unterstützen, also eine Angleichung an die Wachstumsregionen. Tatsächlich sind solche Politiken, beispielsweise im Rahmen des Europäischen Fonds für Regionale Entwicklung, wirksam bei der Reduktion der Unterstützung populistischer Parteien (Albanese et al., 2019; Rodríguez-Pose und Dijkstra, 2020; Gold und Lehr, 2021). Dennoch bleiben periphere Regionen Hochburgen des Populismus. Zielsetzung muss es sein, Entwicklungsperspektiven für abgehängte Regionen zu schaffen. Die reine Subvention von Arbeitsplätzen ist dafür nicht ausreichend. Vielmehr gilt es, auf Basis komparativer regionaler Standortvorteile Potenziale für endogenes Wachstum zu erschließen, beispielsweise als Wohnort für Berufspendler oder als Destination für Tagestourismus. Statt in Wettbewerb zu schnell wachsenden Agglomerationen zu treten, können periphere Regionen so am Erfolg der urbanen Zentren partizipieren.

### Das Thema Migration

Zweifelsfrei haben Anti-Immigrationskampagnen maßgeblich zum länderübergreifenden Erfolg populistischer Parteien beigetragen. Dennoch ist die Forschungslage zum Einfluss tatsächlicher Migration auf das Wahlverhalten uneinheitlich. Immigration kann die Unterstützung populistischer Parteien erhöhen oder verringern, je nach Art der Migration, Charakteristika der Migranten, Charakteristika der aufnehmenden Regionen, etc. Bei kaum einem Politikfeld überlagern sich kulturelle und ökonomische Faktoren, rationale Erwägungen, Ideologie und Psychologie wie beim Thema Migration. Hinzu kommt, dass die Wahrnehmung von Migration nicht immer den tatsächlichen Migrationszahlen entspricht – und diese Wahrnehmung schwankt mit der „Salienz“ von Migration, d. h. deren Sichtbarkeit und Auffälligkeit (Colussi et al., 2021).

Auch wenn die Einflussmöglichkeiten begrenzt sind, gibt es doch auch hier Ansatzpunkte für Wirtschaftspolitik. Zunächst erhöht ökonomische Unsicherheit die Anfälligkeit für Anti-Immigrationsrhetorik. Weiterhin kann Immigration den Wettbewerb auf den lokalen Arbeitsmärkten erhöhen, vor allem für Geringqualifizierte. Hier greifen die oben diskutierten Politikmaßnahmen. Schließlich erhöht Migration auch den Wettbewerb um öffentlich bereitgestellte Güter, zumindest in der Wahrnehmung vieler Menschen (Alesina et.al., 1999; Dahlberg et al., 2012). Das Angebot öffentlicher Güter, beispielsweise von Kinderbetreuungseinrichtungen, sollte daher auf zunehmende Immigration reagieren. Der bloßen Wahrnehmung eines verstärkten Wettbewerbs kann durch Politikdesign effektiv begegnet werden. Beispielsweise lassen sich Fördermaßnahmen für Migranten in allgemeinere Programme zur Förderung benachteiligter Personen integrieren. Dem Eindruck eines Nullsummenspiels zwischen Ursprungsbevölkerung und Migranten lässt sich so entgegenwirken.

Wirtschaftspolitische Maßnahmen, die nicht bloß auf die Kompensation von Verteilungseffekten ausgelegt sind, sondern Entwicklungsperspektiven für benachteiligte Individuen und Regionen bieten, beschränken das populistische Wählerpotenzial und erhöhen die Akzeptanz wohlfahrtsteigernder Entwicklungen. Der Erfolg solcher Politiken hängt auch davon ab, dass Wähler:innen über verlässliche Informationen verfügen, um alternative politische Konzepte und deren Wirkungen evaluieren zu können. Der Erfolg populistischer Parteien beruht aber gerade auf einer Kommunikationsstrategie, die die verlässliche Information über politische Sachverhalte erschwert. Unabhängig von Wahlergebnissen liegt hier eine der größten Herausforderungen durch den Populismus, nicht nur für politische Parteien: der bewussten Fehlinformation der Bevölkerung durch geeignete Kommunikationsstrategien entgegenzuwirken.

Robert Gold forscht am Kieler Institut für Weltwirtschaft (IfW) zur Politischen Ökonomie der Globalisierung und des technologischen Wandels.


Literatur

Albanese, G., G. Barone und G. de Blasio (2019), Populist Voting and Losers’ Discontent. Does Redistribution Matter?, Marco Fanno Working Paper, 0239.
Alesina A Baqir R Easterly W Public Goods and Ethnic Divisions The Quarterly Journal of Economics 1999 114 4 1243 1284 10.1162/003355399556269
Bode E Gold R Adult Training in the Digital Age. Economics: The Open-Access Open-Assessment E-Journal 2018 12 1 14
Colussi, T., I. Isphording und N. Pestel (2021), Minority Salience and Political Extremism, American Economic Journal: Applied Economics, im Erscheinen.
Dahlberg M Edmark K Lundqvist H Ethnic Diversity and Preferences for Redistribution Journal of Political Economy 2012 120 1 41 76 10.1086/665800
Dippel, C., R. Gold und S. Heblich (2015), Globalization and Its (Dis-)ontent. Trade Shocks and Voting Behavior, NBER Working Paper, 21812.
Dippel, C., R. Gold, S. Heblich und R. Pinto (2021), Trade Effects on Workers and Voters, The Economic Journal, im Erscheinen.
Fetzer T Did Austerity Cause Brexit? American Economic Review 2019 109 11 3849 3886 10.1257/aer.20181164
Fetzer, T. und R. Gold (2019), What Drives Populist Votes? Recent Insights and Open Questions. Forum for a New Economy Working Paper 01/2019.
Gold, R. und J. Lehr (2021), Paying Off Populism. How Regional Policy Affects Voting Behavior, im Erscheinen.
Glitsch, J. (2021), Globalization, Populism, and the Welfare State. Compensating the Left-Behind, mimeo.
Guriev, S. und E. Papaioannou (2021), The Political Economy of Populism, Journal of Economic Literature, mimeo.
Rodríguez-Pose, A. und L. Dijkstra (2020), Does Cohesion Policy Reduce EU Discontent and Euroscepticism?, Papers in Evolutionary Economic Geography, 2040.
Rodrik, D. (2020), Why Does Globalization Fuel Populism? Economics, Culture, and the Rise of Right-Wing Populism, NBER Working Paper, 27526.
Rodrik D Populisms and the Economics of Globalization Journal of International Business Policy 2018 1 12 33 10.1057/s42214-018-0001-4
